# Causes of Death in Children Aged < 15 Years in the Inner Mongolia Region of China, 2008-2012

**DOI:** 10.5539/gjhs.v8n9p76

**Published:** 2015-10-29

**Authors:** Ying Wang, Maolin Du, Zhihui Hao, Hairong Zhang, Qing Zhang, Wenli Hao, Lei Xi, Juan Sun

**Affiliations:** 1 Inner Mongolia Medical University, Hohhot, China; 2 Inner Mongolia People’s Hospital, Hohhot, China; 3 Inner Mongolia Medical University Affiliated Hospital, Hohhot, China

**Keywords:** <15 years old, perinatal deaths, injuries

## Abstract

The objective of our study was to identify the causes of death in children <15 years of age in Inner Mongolia and to examine the age-specific causes of death. Study data from 2008–2012 were obtained from the Death Registry System that is maintained by the Inner Mongolia Centers for Disease Control and Prevention. The mortality rate (per 100,000) for children <15 years of age was calculated and stratified by age in different years. We computed the proportion of age-specific causes of death for children <15 years that occurred between 2008 and 2012 across eight monitoring points in Inner Mongolia. We used a log-linear model to analyze the year and age effects on childhood mortality. From 2008-2012, the standardized mortality of children <15 years of age was 42.78/100,000. The mortality rate was not significantly different from 2008 to 2012 (p>0.05); the mortality rate was the highest in the <1-year age group (p<0.05); and the mortality rate of the <1-year age group was higher in 2012 compared to that in 2009 (p<0.05), 2010 (p<0.05), and 2011 (p<0.05). In children aged 1-14 years, the leading cause of death was injuries, among which transport accident injuries were the most prevalent. To reduce the childhood mortality rate in Inner Mongolia, China, we should focus on the prevention of perinatal deaths in infants <1 year of age and on the prevention of transport accident injuries among older children (1-14 years).

## 1. Introduction

Trends in the causes of child deaths serve as important global health predictors to guide efforts to reduce childhood mortality (Black et al., 2010; Johnson, Liu, Walker, & RE, 2010). For low-income and middle-income countries, these indicators are particularly important for assisting child health policy development and scarce resource allocation.

The infant (<1 year) mortality rate is always highest in children <15 years of age (Chen, 2013). Among the many indexes of public health, the infant mortality rate is one of the most important indicators of the regional, national, and population status in various fields, including health, demographics, society, economy, biology, and culture. The infant (<1 year) mortality rate is therefore a very important index of a country’s status, health and development. According to data from the World Health Organization (WHO), the infant (<1 year) mortality rate was 5,310, 4,000, 1,520, and 580 per 100,000 in low-income, lower-middle-income, upper-middle-income, and high-income country, respectively, in 2015 (WHO, 2015). Decreases in infant mortality rate are the result of improvements in infant health and survival, which positively influence the overall state of a nation’s public health (Chung, Choi, & Bae, 2011).

Injuries are a leading cause of death in children worldwide (Peden, Mcgee, & Sharma, 2002). More than 5,000 children die from injury each year in the European Union (EU), accounting for 32% of all deaths of children between 1 and 14 years of age (Strukcinskiene, 2011). Road traffic injuries are the leading cause of death among children aged 0–14 years, causing 34% of childhood (0–14 years) injury deaths annually (Vincenten, 2004). In developed countries, prevention strategies have resulted in steep declines in mortality rates due to unintentional injuries (Pearson, & Stone, 2009; RoSPA, 2007). In contrast, although a significant percentage of childhood morbidity and mortality is attributed to traffic accidents (Blum, Gates, & Farah, 2011; Debata, Deswal, & Kumath, 2014), health initiatives in developing countries are primarily focused on the common causes of childhood death, including diarrhea and malnutrition (Collaborators, 2010).

To date, no study has evaluated the deaths in children < 15 years of age in Inner Mongolia. Therefore, the aim of this study was to examine the deaths in children < 15 years of age and determine the age-specific contribution of each cause of death.

## 2. Methods

Data from 2008 to 2012 were obtained from the Death Registry System (DRS), which is in charge of the Chinese Ministry of Health and run by the Inner Mongolia CDC. The DRS uses a multistage cluster probability sampling strategy with stratification according to eastern, central and western China; the local gross domestic product and proportion of rural dwellers; and the total population of local areas (Xin et al., 2014). The eight monitoring points from the death registry system are Yakeshi City, Kailu County, Bairin Youqi Sonid Youqi, Muslims District, Tumd Youqi, Ejin Horo Qi, and Linhe District. The eight monitoring points are located in the eastern, middle and western regions of Inner Mongolia (Li et al., 2014). The annual average population of the eight monitoring points was 2.4 million, accounting for approximately 10% of the total population of Inner Mongolia (Xin et al., 2014). The annual midyear population figures from 2008-2012 were obtained from the CDC of Inner Mongolia in order to calculate the age-specific mortality and annual mortality per 100,000 persons (Guo et al., 2015). The cause of death was coded according to the International Classification of Disease-10th Revision (ICD-10). The ICD-10 codes that were used in this study were as follows: V01–Y98: Injuries (External causes of morbidity and mortality); P00–96: Perinatal conditions (conditions arising during the perinatal period); Q00–99: Congenital abnormalities (congenital malformations, deformations, and chromosomal abnormalities); C00-D48: and Neoplasms; I00–99: Diseases of the circulatory system. Other diseases were classified as others (A00- Z99.9 except for V01–Y98, P00–96, Q00–99, C00-D48, I00–99).

The data that were collected from the DRS included information of sex, age, ethnicity, place of death, cause of death, and regions, including rural and urban areas. Urban area refers to the cases in areas surrounding a city and under the jurisdiction of an urban government before they die, whereas rural area refers to a village or pastoral area (Hu et al., 2014; Guo et al., 2015).

Sex, age, regions, ethnicity and place of death were chosen as indicators to assess the demographic characteristics of death < 1 or 15 years of age. The potential years of life lost (PYLL) for children < 1 or 15 years of age were calculated. The PYLL are used to emphasize premature mortality by estimating the average number of years a person would have lived had he or she not died prematurely. PYLL = Σ (a_i_×d_i_), a_i_: years of life lost in each age group, and d_i_: the number of deaths in each age group. We computed the proportionate causes of death at in eight monitoring points across Inner Mongolia from 2008-2012. We also computed the proportionate of causes of death in sex and region. The difference in the proportionate of causes of death was tested using Pearson’s chi-square test. The crude mortality and standardized mortality (per 100,000) for children <15 years of age were calculated in age-specific groups for difference years. Crude mortality: number of deaths/number of midyear population. Standardized mortality: ΣN_i_P_i_/N, ΣN_i_P_i_. The sum of expected the death of the “standard population” N: the number of the standard population. A log-linear model (multi-factored contingency table) was used. The factors that were involved in the log-linear model included year and age-specific group. A map was provided to show the eight monitoring points. All of the statistical analyses were performed using Microsoft Excel and SPSS statistical software (version 13.0), and the level of significance was set at P < 0.05.

## 3. Results

The crude mortality of children < 1 or 15 years of age was 282.90/100,000 and 42.72/100,000, respectively from 2008-2012. The standardized mortality was 285.60/100,000 and 42.78/100,000, respectively. The PYLL were 25,993 and 52,253 person years, respectively.

Table 1 shows the demographic characteristics of age-specific deaths of children at eight monitoring points in Inner Mongolia between 2008 and 2012. The Han nationality had the highest percentage of deaths compared to those of the other nationalities, and the percentage of deaths in boys was 1.5 times higher than that in girls. Deaths in urban areas were more frequent than in rural areas, although the difference was small. Most of the deaths occurred in a hospital setting.

**Table 1:** Demographic characteristics of age-specific deaths of children at eight monitoring points in Inner Mongolia between 2008 and 2012

	<15 y		<1 y	
	
	n	%	n	%
**Sex**				
Male	495	62.0	229	61.6
Female	302	37.8	143	38.4
**Nation**				
Han	684	85.7	321	86.3
Mongolian	95	11.9	44	11.8
Other	18	2.3	7	1.9
**Area**				
Urban	419	52.5	199	53.5
Rural	378	47.4	173	46.5
**Place of death**				
Hospital	536	67.2	308	82.8
Home	124	15.5	44	11.8
On the way	68	8.5	13	3.5
Other	69	8.6	7	1.9

The age-specific mortality <15 years of age across the eight monitoring points in Inner Mongolia in 2008 -2012 is shown in Table 2. The results of the log-linear model showed that the mortality rate was not significantly different between different years (p>0.05), the mortality rate was the highest in the <1-year age group (p<0.05), and the mortality rate of the <1-year age group was higher in 2012 than in 2009-2011 (p<0.05).

**Table 2:** Age-specific mortality (1/100,000) <15 years of age at eight monitoring points in Inner Mongolia between 2008 and 2012

	<1 y			1-4 y			5-9 y			10-14 y		
			
	death number	crude mortality	Standardized mortality	death number	Crude mortality	Standardized mortality	Death number	Crude mortality	Standardized mortality	death number	crude mortality	standardized mortality
2008	108	442.0	451.6	19	19.6	19.3	20	19.5	20.0	23	19.7	20.0
2009	62	199.2	199.3	25	20.4	22.8	23	16.9	16.8	41	30.1	30.2
2010	57	183.3	186.2	42	34.2	34.6	27	18.5	19.0	34	26.5	26.8
2011	69	270.6	272.9	37	36.8	37.0	28	23.4	23.8	35	33.3	33.3
2012	76	392.9	393.7	25	31.0	31.1	17	15.8	15.8	29	25.2	25.3

**Figure 1:**
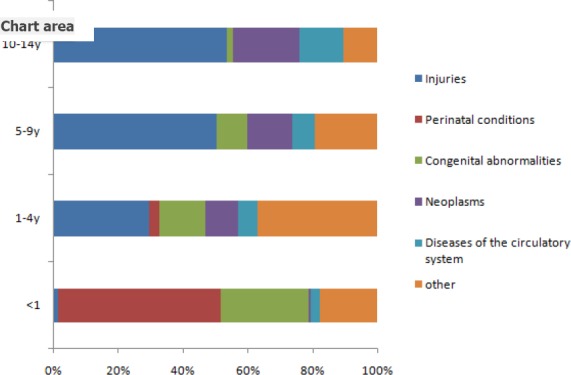
Age-specific proportionate causes of death in children under the age of 15 across eight monitoring points in Inner Mongolia, 2008-2012

Injuries were a significant cause of death of children <15 years of age in Inner Mongolia from 2008 to 2012. Figure 1 shows the proportional contribution of different causes of death in children <15 years of age between age-specific groups based on data that were obtained from eight monitoring points. In the <1-yr age group, deaths due to perinatal conditions comprised the largest proportion of total deaths, at more than 50%, followed by congenital abnormalities. In the 1-14-year overall age group, injuries were the leading cause of childhood deaths, accounting for one-third of the deaths in the 1-4-year age group and half of the deaths in both the 5-9- and 10-14-year age groups. Neoplasms were the second leading cause of childhood deaths across all age groups.

We further calculated the percentage of causes of death in different age-specific groups with different sexes and in different regions. The percentage of causes of death was not significantly different between girls and boys or between urban and rural areas in each age-specific group (p>0.05).

Table 3 indicates the numbers of injury-related deaths across the 4 age-specific groups, obtained from eight monitoring points in Inner Mongolia from 2008 to 2012. The leading cause of injury-related deaths in the overall 1-14-year group was transport accidents injuries, followed by accidental drowning and submersion, falls, suicide, and suffocation. Together, these five causes accounted for more than 80% of injury fatalities in children aged 1-9 years and more than 95% of children between the ages of 10 and 14. For children aged 1-9 years, the proportion of deaths that were caused by transport accidents was higher in rural areas than in urban areas, while transport-accident-related deaths in 10-14-year-old children were more common in urban areas.

**Table 3:** Injury deaths by age group in eight monitoring points in Inner Mongolia, 2008-201

Injure	1-4 y	5-9 y	10-14 y
Transport accidents	21(47.7)	23(39.7)	45(51.7)
Accidental drowning and submersion	2(4.5)	17(29.3)	28(32.2)
Falls	7(15.9)	5(8.6)	5(5.7)
Suicide	0(0)	1(1.7)	4(4.6)
Suffocation	4(9.0)	3(5.2)	2(2.3)
Other	14(31.8)	11(19)	3(3.4)

Data are indicated as n (% of total injury-related deaths).

**Figure 2:**
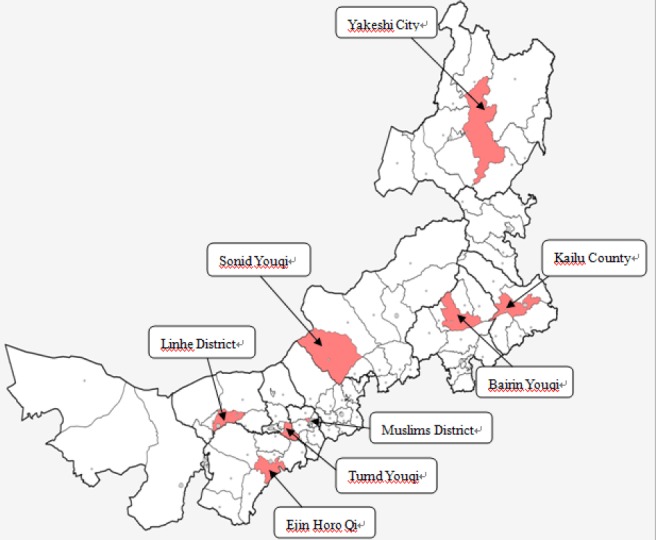
The eight monitoring points in Inner Mongolia

## 4. Discussion

Based on an analysis of data in DRS from Inner Mongolia from 2008 to 2012, the percentage of deaths in children < 15 years of age among all deaths was 1.2%. Compared to WHO data, this percentage is higher than that in Australia (1.1%), Germany (0.4%) and Japan (0.4%) (WHO, 2011). The standardized mortality rate of children < 15 years of age at eight monitoring points in Inner Mongolia from 2008-2012 was 42.78/100,000, which is lower than that in Zhejiang from 2006-2012 (51.41/100,000) (Wang, Chen, & Ze-Lin, 2014) or in Shanghai from 1993-2008 (44.83/100,000) (Hui, Yang, & Yang, 2011). The percentage of deaths in boys is 1.5 times higher than that in girls. This result agrees with a study in Zhejiang (Wang, Chen, & Ze-Lin 2014).

In our study, the standardized mortality rate in infants < 1 year of age was 285.6/100,000, which is nearly four times higher than the mortality rate of the other three age groups combined. However, this rate is lower than the average of OECD (Organization for Economic Cooperation and Development) nations (470/100,000) or the USA (630/100,000) and is comparable to the mortality rate of the same age group among Japanese infants (280/100,000). Japan has the lowest IMR in the world (Chang et al., 2011), indicating that the level of health care for residents of Inner Mongolia, especially maternal and child care, is comparable to that of the nation with the lowest IMR. In the <1-year age group, more than 50% of the deaths occurred as a result of perinatal conditions. Consistent with two studies in Wucheng and Pudong, congenital abnormalities were the second leading cause of death, accounting in our study for 26.88% of the total deaths (Chen, 2013; Hui et al., 2011).

In the 1-14-year age group, injuries were the most common cause of death, which is in agreement with a recent Finnish study (Parkkari, Mattila, Niemi, & Kannus, 2003). Among the different types of injuries, the leading cause of injury-related death among children aged 1-14 years was transport accident injuries. For the 1-9-year age group, the proportion of deaths that were caused by transport accident injuries was higher in rural areas than in urban areas. This finding may result from a combination of a comparative lack of adult supervision in rural areas and not obeying traffic rules (Jiang, 1995) However, half of all road-traffic injury fatalities occurred in the 10-14-year age group, and the proportion of deaths that were caused by transport accident injuries was higher in urban areas than in rural areas. This finding may reflect the increasingly independent nature of children of this age group and the decreasing amount of required adult supervision; additionally, the increased number of vehicles in urban areas results in an elevated numbers of traffic accident casualties. Policies and legislation aimed at reducing the number of pedestrian casualties in Scotland have recently been implemented with the introduction of Home Zones, Safer Routes to School and 20-mph speed limits around schools (Scottish Executive, 2001; Department for Transport, 2007). These policies and legislation should also be applied in Inner Mongolia.

Fall-related injuries were the second most common cause of death for children aged 1-4 years, which underscores the need for parents to be consistently vigilant with respect to their children’s safety. For the 5-14-year age group, accidental drowning and submersion were the second most common causes of injury-related deaths. The proportion of deaths due to accidental drowning and submersions increased with age. Factors that could contribute to accidental drowning and submersion include natural factors (please elaborate on the term natural factors), parental neglect, a large range of holiday activities, risk-taking behavior, lack of awareness of safety measures, and lack of swimming skills (Dai et al., 2012). We observed a number of deaths due to suicide in the 5-14-year age group, and a previous study in Scotland identified suicide as the third leading cause of injury-related deaths in 10-14-year age group (Pearson, & Stone, 2009). This finding underscores the importance of mental health professionals in being made aware of children’s psychological needs during this critical developmental period.

## 5. Conclusion

In summary, we assessed the mortality rates and causes of death in children < 15 years of age in an age-specific manner. Our findings can be of benefit for health care professionals in implementing targeted measures in Inner Mongolia, China, to prevent deaths related to perinatal conditions in children < 1 year of age and injuries in children aged 1-14 years, particularly transport accident injuries.
